# Depressive and anxiety symptoms in postpartum women after recovery from COVID-19: a questionnaire-based observational study

**DOI:** 10.3389/fpubh.2024.1417791

**Published:** 2024-12-19

**Authors:** Chia-Jung Hsieh, Hsiu-Wei Su, Chen-Yu Lee, Chih-Chien Lin, Wei-Chih Chen

**Affiliations:** ^1^Department of Obstetrics, Gynecology and Women's Health, Taichung Veterans General Hospital, Taichung, Taiwan; ^2^Department of Nursing, Taichung Veterans General Hospital, Taichung, Taiwan; ^3^Department of Psychiatry, Taichung Veterans General Hospital, Taichung, Taiwan

**Keywords:** COVID-19, depression, anxiety, postpartum, mental health

## Abstract

**Introduction:**

Previous studies on the association between recovery from Coronavirus Disease 2019 (COVID-19) infection and postpartum depressive and anxiety symptoms had conflicting results. This study aimed to investigate the psychological burdens among postpartum women who had experienced COVID-19 infection during their pregnancies and those who had not.

**Methods:**

This cross-sectional study was conducted at Taichung Veterans General Hospital in Taiwan from April 2022 to January 2023. A total of 113 postpartum women completed self-reported questionnaires, including the Edinburgh Postnatal Depression Scale (EPDS) and General Anxiety Disorder-7 (GAD-7), in the postpartum clinics or wards.

**Results:**

Fifty-four individuals (47%) who experienced COVID-19 infection during their pregnancies and 59 uninfected women completed the questionnaires. The mean EPDS scores were 5.00 ± 4.26 vs. 6.46 ± 4.50 (*p* = 0.09) and the mean GAD-7 scores were 3.17 ± 2.88 vs. 3.69 ± 2.73 (*p* = 0.21). Multivariate linear regression analyses revealed that factors associated with lower EPDS scores included experiencing COVID-19 infection during pregnancy, delivery by expected mode, and neonates not requiring admission to the Intensive Care Nursery (ICN) or Neonatal Intensive Care Unit (NICU). Delivery by expected mode was the only variable associated with a lower GAD-7 score in the multivariate model.

**Conclusion:**

Our study demonstrated that COVID-19 infection during pregnancy was associated with fewer postpartum depressive symptoms based on linear regression analysis, with no significant difference in postpartum anxiety symptoms.

## 1 Introduction

The Coronavirus Disease 2019 (COVID-19) pandemic was declared by the World Health Organization as an international public health emergency on 11 March 2020. Beyond its effects on physical health, fear of COVID-19 infection and the implementation of strict isolation measures produced a negative impact on individuals' psychological wellbeing (1, 2). A previous study revealed that while COVID-19 initially increased anxiety, stress, and depression among patients, these levels significantly decreased following recovery and a better understanding of the disease (3).

Postpartum women are at risk of developing mental health disorders, particularly depression and anxiety. Maternal psychological disorders negatively affect maternal functions and the behavioral, emotional, and cognitive development of infants (4). Around 20% of women during the postpartum period experienced depressive symptoms, while approximately 15% of postpartum women suffered from anxiety symptoms (4, 5). Factors associated with postpartum depression include prenatal depression or anxiety, a personal or family history of depression, unexpected pregnancy, lack of social support, low educational level, recent negative events, and physical illness. Therefore, it is essential to assess women's psychological health during the postnatal period to design targeted interventions based on their specific needs (6, 7).

Studies reported elevated rates of anxiety and depression among pregnant and postpartum women during the COVID-19 pandemic (2, 4, 8–11). A systematic review of 29 articles comparing postpartum women during and before the COVID-19 pandemic revealed an increased prevalence of psychological symptoms among postpartum women during the pandemic (4). Another systematic review of 217 studies yielded similar results, indicating that women in the perinatal period exhibited diverse symptoms of compromised mental health, including depression (24.9%) and anxiety (32.8%), during the pandemic (11). Insufficient social support, social isolation, and concerns about COVID-19 exposure or infection of both themselves and their newborns further increased the psychological burdens of postpartum women (2).

While numerous studies have investigated the mental stress of pregnant and postpartum women during the pandemic, the prevalence of anxiety or depressive symptoms after recovery from COVID-19 infection in perinatal women was less studied. In one study using the International Registry of Coronavirus Exposure in Pregnancy database, women with moderate to severe illness from COVID-19 were more likely to develop anxiety and depressive symptoms. In contrast, the risk was not increased in individuals with no or mild symptoms (12). Another study reported a lower prevalence of depression and anxiety among postpartum women with a positive COVID-19 test compared to those with a negative test; however, the difference was not statistically significant (13). Thus, our study aimed to investigate the psychological burdens among postpartum women with or without COVID-19 infection during their pregnancies.

## 2 Methods

### 2.1 Study design and participants

This cross-sectional study was conducted at a single tertiary center. The study included women giving birth at Taichung Veterans General Hospital in central Taiwan between April 2022 and January 2023. Participants were recruited through a convenience sampling method, with women visiting the hospital for postpartum care invited to join the study. Eligible participants were those who: (a) underwent either COVID-19 antigen test or nucleic acid amplification test during their pregnancies, (b) completed designated self-reported questionnaires, and (c) delivered at a gestational age of at least 24 weeks. Individuals diagnosed with depression, anxiety, or any other psychiatric or personality disorders before pregnancy were excluded from the study. All participants provided informed consent and agreed to participate in the study. The background and objectives of the survey, along with the details of informed consent, were explained on the initial page of the questionnaires. Before initiating the survey, we conducted a sample size calculation using G^*^Power 3.1.9.7, based on assumptions regarding the incidence rates of postpartum depressive symptoms, which ranged from a minimum of 3% to a maximum of 20%. An alpha level of 0.05 and a power of 0.9 were set. Our statistical analysis indicated that a study design involving 59 samples per group, totaling 118 participants, would provide approximately a 90% probability of detecting significant differences. The study was approved by the Institutional Review Board of Taichung Veterans General Hospital with IRB number CG22414A.

### 2.2 Measurement of postpartum depression and anxiety symptoms

We assessed depression and anxiety symptoms with the Chinese versions of the Edinburgh Postnatal Depression Scale (EPDS) and General Anxiety Disorder-7 (GAD-7). The questionnaires were administered by either physicians or nurses in postpartum outpatient clinics or wards. The EPDS, comprising 10 items, is a valid and reliable screening tool widely utilized for assessing postpartum depression (PPD) symptoms. Each item is rated on a 0–3 Likert scale to reflect the intensity of specific depressive symptoms. The total scores range from 0 to 30, and higher scores indicate an elevated risk of experiencing PPD (14, 15). A score of 10 or higher suggests possible postpartum depression and necessitates further evaluation (15). The internal Cronbach's alpha value of the Chinese version of EPDS is 0.87, indicating reliability (16). The GAD-7 is a self-reported instrument consisting of seven items. Participants rate the frequency of statements related to core symptoms of generalized anxiety disorder on a 0–3 Likert scale. The cumulative GAD-7 score ranges from 0 to 21, with higher scores indicating an elevated level of worry or somatic tension (17, 18). The Chinese version of the GAD-7 demonstrates significant reliability, with a Cronbach's alpha value of 0.84. At the optimal cutoff value of 7, it exhibits a sensitivity of 96.8% and a specificity of 56.1% (19).

### 2.3 Statistical analysis

IBM SPSS Statistics, Version 22.0 (IBM Corp., Armonk, NY, USA) was used for statistical analysis. The Mann–Whitney *U*-test was applied to nonparametric continuous data, as the distribution of certain variables, including participants' age, body mass index, delivery age, neonatal birth weight, EPDS total score, and GAD-7 total score, was assessed and found to be non-normal based on the Kolmogorov-Smirnov test. These variables did not meet the assumptions for parametric tests; therefore, the Mann–Whitney *U*-test was used to compare differences between groups. Categorical variables were expressed as percentages and compared by the chi-square test. A *p*-value of < 0.05 was considered statistically significant. Univariate and multivariate linear regression analyses were conducted to identify independent factors influencing EPDS scores and GAD-7 scores as dependent variables. If a *p*-value in the univariate was < 0.2, the variable was included in the multivariate linear regression model. Variables with a *p*-value < 0.05 in the multivariate linear regression model were statistically significant independent variables.

### 2.4 Ethical considerations

In our study, we implemented specific measures to prioritize participant safety. If a participant's EPDS or GAD-7 score exceeded the designated threshold, or if they responded positively to the 10th question of the EPDS (indicating self-harm thoughts), the research team promptly contacted the participant and facilitated a referral to a mental health professional for further assessment and support. This protocol was clearly outlined in the informed consent to ensure participants were aware of the available support during psychological distress.

## 3 Results

During the study period, a total of 113 postpartum women were recruited, and all of them completed the questionnaires. Fifty-four individuals (47%) experienced COVID-19 infection during their pregnancies; all of them had either asymptomatic or mild symptoms, and none of their neonates were reported to have congenital COVID-19 infection. The demographic characteristics of participants were presented in Table 1. The COVID-19 infected group comprised fewer primiparous women as compared to the control (53.7% vs. 72.9%, *p* < 0.05). Apart from that, there were no differences in other characteristics, including maternal age, body mass index, marital status, COVID-19 vaccination status before delivery, educational level, employment status, maternal co-morbidities, gestational age at delivery, mode of delivery, postpartum care in an institution, and whether the mode of delivery was expected. Neonatal outcomes, including newborn birth weight, Apgar score, and admission to the Intensive Care Nursery (ICN) or Neonatal Intensive Care Unit (NICU) after birth, were also presented in Table 1, and no significant differences were observed between the two groups.

**Table 1:** Demographic characteristics of the participants with vs. without COVID-19 infection during pregnancy.

	**COVID-19 infection (*n* = 54)**	**Without COVID-19 infection (*n* = 59)**	***p*-value**
Age (years)	33.17 ± 5.28	33.46 ± 3.98	0.76
Body mass index (kg/m^2^)	26.89 ± 3.36	26.37 ± 4.12	0.28
Delivery age (weeks)	38.29 ± 1.61	38.37 ± 1.61	0.74
Not married^#^, *N* (%)	1 (1.9%)	1 (1.7%)	1.00
Primiparity^+^, *N* (%)	29 (53.7%)	43 (72.9%)	**0.03** ^*^
Educational level			0.27
Less than College^+^, *N* (%)	7 (13.0%)	4 (6.8%)	
College or above, *N* (%)	47 (87.0%)	55 (93.2%)	
Employment^+^, *N* (%)	39 (72.2%)	45 (76.3%)	0.62
Maternal comorbidities			
Hypertension disorder of pregnancy^+^, *N* (%)	7 (13.0%)	6 (10.2%)	0.64
Gestational diabetes^#^, *N* (%)	3 (5.6%)	7 (11.9%)	0.33
^a^Expected mode of delivery^+^, *N* (%)	46 (85.2%)	48 (81.4%)	0.59
Mode of delivery^+^			0.46
Cesarean delivery	21 (38.9%)	19 (32.2%)	
Vaginal delivery	33 (61.1%)	40 (67.8%)	
^b^Postpartum care institution^+^, *N* (%)	38 (70.4%)	45 (76.3%)	0.48
^c^COVID-19 vaccination before delivery^#^, *N* (%)	51 (94.4%)	56 (94.9%)	1.00
Neonatal factors			
Birth weight (gm)	2,997.13 ± 452.81	2,994.25 ± 445.77	0.79
Apgar score (1 min) < 7^+^	13 (24.1%)	12 (20.3%)	0.63
Apgar score (5min) < 7^#^	3 (5.6%)	3 (5.1%)	1.00
ICN or NICU admission	20 (37.0%)	15 (25.4%)	0.18

Continuous variables are presented as mean ± standard deviation and categorical variables as N (%).

^+^Chi-square test.

^#^Fisher's Exact Test.

^*^p < 0.05 was considered statistically significant.

ICN, intermediate care nursery; NICU, neonatal intensive care unit.

^a^Expected mode of delivery: as determined by asking participants in the questionnaire whether the delivery method followed their expectations.

^b^Postpartum care institution: participants whose location for postpartum confinement was at a postpartum care institution.

^c^COVID-19 vaccination before delivery: participants who received two valid doses before delivery.

Table 2 illustrated the comparison of postpartum depressive and anxiety symptoms between the two groups. Women infected with COVID-19 during their pregnancies had similar but slightly lower scores on both the EPDS (5.00 ± 4.26 vs. 6.46 ± 4.50, *p* = 0.09) and GAD-7 (3.17 ± 2.88 vs. 3.69 ± 2.73, *p* = 0.21), although these differences were not statistically significant. The proportion of women scoring higher than the cut-off suggesting PPD (EPDS score ≧10) and anxiety (GAD-7 score ≧7) in the COVID-19 infected group and non-infected group were 18.5% vs. 20.3%, *p* = 0.81 and 14.8% vs. 16.9%, *p* = 0.76. Additionally, women who delivered by the expected mode had significantly lower scores on both the EPDS (5.30 ± 4.29 vs. 8.05 ± 4.48, *p* = 0.01) and GAD-7 (3.20 ± 2.53 vs. 4.63 ± 3.76, *p* = 0.04). Women whose neonates did not require admission to the ICN or NICU had significantly lower EPDS scores (5.13 ± 4.01 vs. 7.17 ± 5.02, *p* = 0.02), but similar GAD-7 scores with no significant difference (3.32 ± 2.68 vs. 3.71 ± 3.09, *p* = 0.49).

**Table 2:** Postpartum depressive and anxiety symptoms among women with and without COVID-19 infection during pregnancy.

	**COVID-19 infection (*n* = 54)**	**Without COVID-19 infection (*n* = 59)**	***p-*value**
Mean EPDS total score	5.00 ± 4.26	6.46 ± 4.50	0.09
EPDS ≧ 10^+^, *N* (%)	10 (18.5%)	12 (20.3%)	0.81
Mean GAD-7 total score	3.17 ± 2.88	3.69 ± 2.73	0.21
GAD-7 ≧ 7^+^, *N* (%)	8 (14.8%)	10 (16.9%)	0.76

Continuous variables are presented as mean ± standard deviation and categorical variables as N (%).

^+^Chi-square test.

EPDS, Edinburgh Postnatal Depression Scale; GAD-7, General Anxiety Disorder-7.

Univariate and multivariate linear regression analyses, conducted to investigate the potential factors influencing EPDS and GAD-7 scores, were demonstrated in Tables 3–6. Factors associated with significantly lower EPDS scores included experiencing COVID-19 infection during pregnancy [*B* = −1.60, 95% CI (−3.19, −0.01), *p* = 0.048], delivery by expected mode [*B* = −2.26, 95% CI (−4.39, −0.12), *p* = 0.038], and neonates not requiring admission to the ICN or NICU [*B* = −1.97, 95% CI (−3.71, −0.23), *p* = 0.027; Table 4]. Delivery by expected mode [*B* = −1.38, 95% CI (−2.75, 0.00), *p* = 0.049] was the only variable associated with significantly lower GAD-7 scores in the multivariate model (Table 6).

**Table 3:** Univariate linear regression analyses of individual variables related to Edinburgh Postnatal Depression Scale scores.

**Variable**	***n***	***B***	**95% CI**	**β**	***t***	***p-*value**
**COVID-19 infection during pregnancy**	54	−1.46	[−3.09, 0.18]	−0.17	−1.76	**0.080**
Primiparity	72	−0.11	[−1.83, 1.62]	−0.01	−0.12	0.902
Educational level (college or above)	102	−0.06	[−2.86, 2.73]	−0.04	−0.46	0.964
Maternal comorbidities	49	0.49	[−1.18, 2.16]	0.06	0.59	0.559
^**a**^ **Expected mode of delivery**	94	−2.75	[−4.91, −0.60]	−0.23	−2.53	**0.013** ^*^
^b^Postpartum care institutions	83	1.17	[−0.69, 3.04]	0.12	1.25	0.215
**Neonate non-admission to ICN or NICU**	78	−2.04	[−3.79, −0.29]	−0.21	−2.31	**0.023** ^*^

p < 0.2 was used to select variables for multivariate analysis.

^*^p < 0.05 was considered statistically significant.

CI, Confidence interval for B; ICN, Intermediate care nursery; NICU, Neonatal intensive care unit.

^a^Expected mode of delivery: as determined by asking participants in the questionnaire whether the delivery method followed their expectations.

^b^Postpartum care institution: participants whose location for postpartum confinement was at a postpartum care institution. The bold values indicate variables where p < 0.2, which were used for selecting variables for multivariate analysis.

**Table 4:** Multivariate linear regression analyses of individual variables related to Edinburgh Postnatal Depression Scale scores.

**Variable**	***B***	**95% CI**	**β**	***t***	***p-*value**
COVID-19 infection during pregnancy	−1.60	[−3.19, −0.01]	−0.18	−2.00	0.048^*^
^a^Expected mode of delivery	−2.26	[−4.39, −0.12]	−0.19	−2.10	0.038^*^
Neonate non-admission to ICN or NICU	−1.97	[−3.71, −0.23]	−0.21	−2.25	0.027^*^

${\text{R}}_{\text{adj}}^{2}$ = 0.133 (N = 113).

^*^p < 0.05 was considered statistically significant.

CI, Confidence interval for B; ICN, Intermediate care nursery; NICU, Neonatal intensive care unit.

^a^Expected mode of delivery: as determined by asking participants in the questionnaire whether the delivery method followed their expectations.

**Table 5:** Univariate linear regression analyses of individual variables related to General Anxiety Disorder-7 scores.

**Variable**	***n***	***B***	**95% CI**	**β**	***t***	***p-*value**
COVID-19 infection during pregnancy	54	−0.53	[−1.57, 0.52]	−0.09	−1.00	0.319
Primiparity	72	0.16	[−0.93, 1.25]	0.03	0.29	0.774
Educational level (college or above)	102	−0.11	[−1.88, 1.66]	−0.03	−0.35	0.899
Maternal comorbidities	49	−0.17	[−1.23, 0.89]	−0.03	−0.32	0.753
^**a**^ **Expected mode of delivery**	94	−1.43	[−2.81, −0.05]	−0.19	−2.06	**0.042** ^*^
^**b**^ **Postpartum care institutions**	83	0.83	[−0.35, 2.01]	0.13	1.39	**0.166**
Neonate non-admission to ICN or NICU	78	−0.39	[−1.53, 0.74]	−0.07	−0.69	0.492

p < 0.2 was used to select variables for multivariate analysis.

^*^p < 0.05 was considered statistically significant.

CI, Confidence interval for B; ICN, Intermediate care nursery; NICU, Neonatal intensive care unit.

^a^Expected mode of delivery: as determined by asking participants in the questionnaire whether the delivery method followed their expectations.

^b^Postpartum care institution: participants whose location for postpartum confinement was at a postpartum care institution. The bold values indicate variables where p < 0.2, which were used for selecting variables for multivariate analysis.

**Table 6:** Multivariate linear regression analyses of individual variables related to General Anxiety Disorder-7 scores.

**Variable**	***B***	**95% CI**	**β**	***t***	***p-*value**
^a^Expected mode of delivery	−1.38	[−2.75, 0.00]	−0.18	−1.99	0.049^*^
^b^Postpartum care institutions	0.76	[−0.40, 1.93]	0.12	1.30	0.196

${\text{R}}_{\text{adj}}^{2}$ = 0.042 (N = 113).

^*^p < 0.05 was considered statistically significant.

CI, Confidence interval for B; ICN, Intermediate care nursery; NICU, Neonatal intensive care unit.

^a^Expected mode of delivery: as determined by asking participants in the questionnaire whether the delivery method followed their expectations.

^b^Postpartum care institution: participants whose location for postpartum confinement was at a postpartum care institution.

## 4 Discussion

In this single-center, cross-sectional study, women who experienced COVID-19 infection during their pregnancies, compared to the non-infected group, demonstrated fewer depressive symptoms and no significant difference in anxiety symptoms in the postpartum period, as determined by a linear regression model.

Sustained fear of COVID-19 during pregnancy acts as a stressor on mental health. Pregnant individuals worry about the pervasive influence of COVID-19 and public policies that are intended to mitigate its spread, such as quarantine and isolation. Moreover, they are concerned about the potential impact of COVID-19 infection on their health or their fetus. Giesbrecht et al. (20) reported that heightened fear of COVID-19 was linked to higher odds of depression [adjusted odds ratio (aOR) = 1.75, *p* < 0.001, 95% CI 1.66–1.85] and anxiety (aOR = 2.04, *p* < 0.001, 95% CI 1.94–2.15). This indicates that persistent concern about COVID-19 during pregnancy is associated with elevated levels of depressive and anxiety symptoms.

Our study showed that women infected with and recovered from COVID-19 during their pregnancies had fewer depressive symptoms. This result may seem incomprehensible, and we offer three possible explanations. Firstly, it has been demonstrated that most pregnant women infected with COVID-19 manifest no or mild symptoms, which are not associated with adverse pregnancy and neonatal outcomes (21, 22). Secondly, the risk of newborns having congenital COVID-19 appears uncommon (23, 24). Lastly, prior infection provides protection against reinfection and affords longer-lasting immunity against hospitalization or severe illness (25). In our study population, all pregnant women with COVID-19 infection were either asymptomatic or presented with mild disease, and none of them had babies with congenital infection. Compared to women not infected with COVID-19, the recently infected individuals had less fear as they perceived themselves as having stronger immunity, and they were less troubled by the uncertainty of catching new COVID-19 and its consequences on maternal and fetal outcomes. Therefore, participants with a history of COVID-19 infection in our study may exhibit fewer depressive symptoms due to the explanations mentioned above.

We found that delivering via the expected mode is an important factor associated with lower EPDS and GAD-7 scores in the multivariate model. Dekel et al. assessed the relationship between mode of delivery and prenatal and postnatal mental health. They reported that women who underwent surgical or instrumental deliveries exhibited higher levels of depression and anxiety symptoms compared to those who had natural deliveries. Unanticipated cesarean sections also caused elevated levels of childbirth-related posttraumatic stress disorder (PTSD) symptoms (26). Houston et al. conducted a longitudinal study to explore the relationship between delivery-mode preferences and postpartum depression. Their study revealed that those with a strong antepartum preference for vaginal delivery who ultimately delivered by cesarean were at an increased risk for depression in the early postpartum period (27). The results of our study are consistent with these findings, indicating that delivering via planned mode is associated with lower levels of depression and anxiety symptoms.

Having a newborn that did not require admission to the ICN or NICU was also an important factor associated with a lower EPDS score in our multivariate model. Lin et al. enrolled 1197 postpartum women from a baby-friendly hospital in Taiwan to examine the risk and protective factors for PPD. They found that admission to the ICN or NICU significantly increased the risk of PPD, with an adjusted odds ratio of 2.29. The unexpected admission of neonates to these units often triggers negative emotions in their mothers, such as uncertainty about the child's health, feelings of inadequacy in caring for their newborn, reduced maternal bonding, and even financial stress (14).

The relationship between parity and maternal mental health remains inconclusive. While several studies describe primiparity as a risk factor for developing PPD (28, 29), others indicate that parity is not associated with maternal PPD symptoms (30, 31). In our study, we did not find primiparity to be associated with maternal psychological burden.

In Taiwan, “doing-the-month,” the traditional Chinese postpartum confinement practice, is regarded as an essential intervention for supporting maternal recovery during the postpartum period. Hung et al. indicate that “doing-the-month” in postpartum nursing centers reduces postpartum stress and enhances the general health of postpartum women. This improvement is attributed to the comprehensive care provided by these centers, which includes maternal and infant care, dietary supplements, and increased opportunities for rest (32). Therefore, our study incorporated postpartum care in nursing homes as a variable to assess its impact on postpartum mental health; however, linear regression analyses revealed no significant correlation.

## 5 Limitations

Firstly, the research was conducted within a single tertiary medical center in central Taiwan, which may lead to a sampling bias because individuals with medical complications were more likely to be included. When interpreting the findings of the study, it is important to consider potential selection bias and cultural differences. Secondly, this cross-sectional study is susceptible to recall bias and confounding factors. Thirdly, we did not perform repeated measures to differentiate participants with temporary or persistent anxiety/depressive symptoms. All participants with psychological distress were identified by self-reported questionnaires, lacking a rigorous diagnostic psychiatric interview or evaluation. Fourthly, there may be other unmeasured factors. Evidence from the literature highlights several additional factors that may influence mental health outcomes, such as social support, breastfeeding, skin-to-skin contact after birth, and maternal-infant bonding. These factors could have been affected during the COVID-19 pandemic and might influence postpartum mental health (15, 33). Future studies and more comprehensive assessments are needed to evaluate these unmeasured factors affecting postpartum mental health. Finally, the small sample size may have resulted in underpowered statistical comparisons for certain variables.

## 6 Conclusions

We demonstrated that COVID-19 infection during pregnancy was associated with fewer postpartum depressive symptoms based on linear regression analysis, while no significant difference was observed in postpartum anxiety symptoms.

In future research, multi-center studies with larger sample sizes are recommended to reduce selection bias and increase statistical power. Longitudinal studies are also necessary to provide insights into the persistence of anxiety and depressive symptoms over time. Additionally, the use of rigorous diagnostic psychiatric interviews could enhance the validity of self-reported psychological distress and enable a more comprehensive examination of the long-term effects of the post-COVID-19 pandemic on postpartum mental health.

## Data Availability

The raw data supporting the conclusions of this article will be made available by the authors, without undue reservation.
